# Self-Supervised Representation Learning from Ventilator Waveforms: A Pilot Feasibility Study

**DOI:** 10.21203/rs.3.rs-11325296/v1

**Published:** 2026-10-14

**Authors:** Seibi Kobara, Md Hassanuzzaman, Vijay Krishnamoorthy, Tetsu Onuma, Mihai V. Podgoreanu, Krista L. Haines, Suresh M. Agarwal, Rishikesan Kamaleswaran

## Abstract

**Rationale:** Modern ventilators generate high-frequency pressure and flow waveforms that contain respiratory mechanics, patient respiratory effort, and patient–ventilator interactions. However, these waveform data remain underutilized for representation learning. **Objective:** We conducted a pilot feasibility study to evaluate whether self-supervised representation learning can extract clinically relevant respiratory information from ventilator waveforms. **Methods:** We developed a self-supervised representation learning framework for mechanical ventilation waveforms using airway pressure and flow signals. The model was pretrained on unlabeled waveform segments over 255,355 breaths spanning 197.1 hours of mechanical ventilation across 115 patients and learned latent respiratory representations. Frozen embeddings were subsequently evaluated on held-out patients using downstream tasks, including ventilator mode classification, prediction of respiratory physiological variables, and reconstruction of waveforms. **Measurements and Main results:** Pretrained embeddings enabled recovery of ventilator modes, achieving a bootstrap-derived median balanced accuracy of 0.791 (95% CI: 0.715, 0.858) and macro-F1 score of 0.765 (95% CI: 0.682, 0.839). The learned representations also supported the prediction of respiratory physiological variables, including respiratory rate and positive end-expiratory pressure. Reconstruction of unseen waveforms achieved a median Pearson correlation of 0.801 (95% CI: 0.727, 0.870) for pressure waveforms and 0.783 (95% CI: 0.709, 0.838) for flow waveforms between the reconstructed and original waveforms. **Conclusions:** This pilot study demonstrates the feasibility of self-supervised representation learning from ventilator waveforms. Our framework introduces ventilator waveforms as a potentially useful data modality in critical care and establishes a foundation for future investigations of patient–ventilator asynchrony, respiratory state monitoring, and optimization of liberation from mechanical ventilation.

## Impact of research statement

We developed a self-supervised representation learning framework for mechanical ventilation using ventilator waveforms. Our framework learns compact respiratory embeddings that preserve physiological information for diverse downstream tasks while enabling waveform reconstruction. This work establishes a computational foundation for future respiratory digital twins, intelligent ventilator monitoring, and precision mechanical ventilation in critical care.

## Introduction

Mechanical ventilation is a life-sustaining therapy used to support patients with respiratory failure. During mechanical ventilation, clinicians continuously monitor airway pressure, flow, and volume waveforms to assess respiratory mechanics, patient-ventilator interactions, and readiness for liberation from ventilatory support. Appropriate ventilator management is essential, as prolonged mechanical ventilation is associated with adverse outcomes, including ventilator-induced lung injury[1], ventilator-associated pneumonia[2], diaphragm dysfunction[3], and delayed recovery[4]. Consequently, protective ventilation strategies and timely liberation from mechanical ventilation have become major goals of critical care practice[5,6].

Modern ventilators generate high-frequency waveform data in real time. These waveforms contain detailed information regarding respiratory mechanics, ventilator settings, patient respiratory effort, and patient-ventilator synchrony. Clinicians routinely interpret waveform abnormalities—double trigger, inspiratory pressure sagging, and beaking at the end of inspiration—to optimize ventilator settings and improve patient-ventilator interaction. Although ventilator waveforms have been increasingly analyzed using computational approaches, existing methods primarily rely on rule-based interpretation or are developed for specific prediction tasks, such as asynchrony detection or ventilator mode classification[7–14]. Consequently, they do not learn transferable representations that characterize evolving respiratory physiology over the course of mechanical ventilation.

Recent advances in foundation models have demonstrated that self-supervised learning can extract robust latent representations from large-scale unannotated data. Foundation models have shown remarkable success across natural language processing, electronic health records, and biomedical data[15,16], enabling transfer learning and improved performance across diverse downstream tasks. By learning generalizable representations directly from raw data, foundation models provide an opportunity to move beyond handcrafted features and task-specific modeling[15]. Ventilator waveforms contain rich physiological information reflecting respiratory mechanics, patient–ventilator interactions, respiratory effort, and ventilator settings. Ventilator waveforms represent a promising yet largely unexplored modality for foundation-model development.

In this pilot feasibility study, we sought to develop a self-supervised representation-learning framework for mechanical ventilation waveforms that can support multiple downstream applications, including characterization of patient-ventilator interactions. As an initial evaluation of this framework, we focused on establishing whether clinically relevant ventilator and respiratory characteristics can be learned and preserved within representations and chose ventilation mode, respiratory rate, and positive end-expiratory pressure (PEEP) as proof-of-concept evaluation tasks. These variables were not intended as clinical prediction targets but rather as objective measures for establishing the feasibility of respiratory representation learning before extending the framework to more complex applications. We additionally evaluated whether ventilator waveforms could be reconstructed from the derived respiratory embeddings. These findings introduce ventilator waveforms as a potentially valuable data modality to evaluate respiratory latent state, providing future studies of respiratory state monitoring, simulation of therapeutic interventions, and optimization of liberation from mechanical ventilation.

## Methods

We used publicly available mechanical ventilation waveform data collected from 145 critically ill patients admitted to intensive care units at the University of California Davis Medical Center[12]. The publicly available dataset does not include detailed clinical characteristics of the cohort or clinical information associated with the ventilation data. The dataset contained airway pressure and flow waveforms sampled at 50 Hz using Puritan Bennett ventilators and included multiple ventilation modes: volume-controlled ventilation (VCV), pressure-controlled ventilation (PCV), pressure support ventilation (PS), continuous positive airway pressure (CPAP), and proportional-assist ventilation (PAV). In total, the dataset comprised 324,839 breaths spanning 247.5 hours of waveform recordings. The number of breaths and total ventilation hours for each ventilation mode is provided in Supplementary Table E1. Ventilator mode labels and breath-level respiratory parameters were extracted from the accompanying metadata and used for downstream evaluation tasks.

## Ventilation waveform representation learning

We developed Ventilation Waveform Transformer (VentWaveT), a self-supervised representation learning framework for ventilator waveform representation learning. Clinically, respiratory cycles vary substantially in duration, and breath boundaries are often ambiguous, making breath segmentation difficult and limiting real-time clinical application. To address these challenges, we adopted a segmentation-free strategy. During pretraining, 100,000 waveform segments, each consisting of 750 time points (15 seconds at 50 Hz), were randomly sampled from continuous recordings. Consequently, each segment could contain partial breaths, complete breaths, or multiple respiratory cycles.

Pressure and flow waveforms were processed using a one-dimensional convolutional neural network (1D-CNN) encoder to extract 30 tokens and 128 latent respiratory features, and convolutional positional encodings across tokens were incorporated to preserve temporal information.

During pretraining, 30% of waveform tokens were randomly masked, and the model was trained to reconstruct the masked signals using a self-supervised reconstruction objective. Following pretraining, the encoder was frozen and used to generate respiratory embeddings for downstream tasks. The token representations were averaged across token dimension to generate 128-dimantional respiratory embeddings. This aggregation was intentionally designed to provide a flattened representation suitable for future integration with longitudinal multimodal data and intervention simulation. The detailed model specification is provided in the Online Supplement.

### Downstream evaluation

To assess whether VentWaveT learned clinically relevant respiratory representations, we evaluated the pretrained embeddings on multiple downstream tasks, including ventilator mode classification and prediction of respiratory rate and PEEP. At the bedside, these variables are already available before inspecting ventilator waveforms. They were selected as evaluation tasks because they provide objective measures of whether the learned embeddings capture clinically relevant ventilator and physiological information. Successful recovery of these variables indicates that the latent representations encode fundamental respiratory characteristics. This provides a foundation for future investigations into whether the same representations also capture more complex respiratory phenomena, such as patient-ventilator asynchrony, residual respiratory impairment (e.g., end-expiratory flow limit), and respiratory muscle fatigue.

First, ventilator mode classification was performed using multinomial logistic regression models applied to frozen embeddings. Because ventilator mode labels were available at the breath level, individual respiratory cycles were cyclically repeated or truncated to 750 time points prior to embedding extraction. Classification performance was evaluated on previously unseen patients using accuracy, precision, recall, F1-score, and specificity. To visualize the structure of the learned respiratory representations, pretrained embeddings from the training dataset were projected into two dimensions using Uniform Manifold Approximation and Projection (UMAP). To identify waveform regions contributing to ventilator mode classification, embedding feature-attribution maps were computed and visualized on representative waveforms. A detailed description of the UMAP analysis is provided in the Online Supplement.

Second, we evaluated whether the learned representations captured quantitative respiratory physiology by predicting respiratory rate (RR) and positive end-expiratory pressure (PEEP) using linear regression models applied to frozen embeddings. RR was manually calculated from individual breath duration as 60 divided by breath duration in seconds, whereas PEEP values were obtained from observed values annotated by the authors of the original dataset[12]. Regression performance was assessed using mean absolute error (MAE) and Pearson correlation coefficient ( $\rho$ ).

### Ablation experiments

We performed a series of ablation experiments to assess whether VentWaveT learned physiologically meaningful respiratory representations and whether these representations were readily accessible using simple downstream models.

First, to determine whether the pretrained embeddings encoded information beyond the latent distribution itself, we compared VentWaveT embeddings with random embeddings generated by independently sampling each latent dimension from a Gaussian distribution parameterized by the feature-wise mean and standard deviation of the pretrained embeddings. This procedure preserved the marginal distribution of the latent space while removing information encoded from ventilator waveforms.

Second, to assess whether nonlinear readout was necessary to extract information from the learned representations, we performed an exploratory ablation analysis evaluating a shallow multilayer perceptron (MLP) and an extreme gradient boosting (XGBoost) classifier[17] with prespecified parameters alongside the primary linear models. Detailed model specifications of the MLP and XGBoost models are provided in the Online Supplement.

### Benchmarking against general-purpose time-series foundation models

To evaluate the quality of the learned respiratory representations, VentWaveT was benchmarked against two state-of-the-art general-purpose time-series foundation models (TSFMs), including MOMENT[18] and Chronos-2[19]. Both models are encoder-only architectures that generate contextual embeddings from time-series data. A detailed model specification and evaluation procedures are provided in the Online Supplement.

### Reconstruction of waveforms from unseen respiratory embeddings

We further developed a transformer-based decoder to reconstruct ventilator waveforms from the respiratory embeddings learned by VentWaveT. Because VentWaveT intentionally compresses each 15-second, breathing cycle-agnostic waveform segment into a compact embedding, the decoder provides a means to recover physiologically plausible waveforms from these latent representations. Beyond demonstrating the physiological information preserved in the embeddings, this reconstruction framework establishes a foundation for future applications, including the realization of physiologically plausible ventilator waveforms from counterfactual respiratory state simulations.

To evaluate reconstruction performance, we first prepared 100 randomly shifted waveforms for each breath and extracted respiratory embeddings from unseen held-out patients. The embeddings were used to reconstruct waveforms using the trained decoder. Because the respiratory embeddings were designed to represent waveform morphology, rather than absolute temporal phase, each reconstructed waveform was aligned with its corresponding original waveform by identifying the temporal shift that minimize mean squared error. Reconstruction performance was subsequently evaluated between the aligned reconstruction and the original waveform. A detailed description of decoder training and performance evaluation procedures is provided in the Online Supplement.

## Statistical analysis

To evaluate the model performance of the mode classification and prediction of RR and PEEP, 1,000 mode-stratified patient-level bootstrap resamples were used to estimate the means with 95% confidence intervals (CIs), median, and interquartile ranges (IQRs), while the proportion of true labels in each bootstrap sample should exceed 0.1%. Because the number of breaths vary considerably across ventilation mode and patients, the bootstrap resampling above could occasionally result in extremely sparse representation of a ventilation mode. This threshold was therefore applied to exclude class-specific performance estimates based on such extremely sparse true-label representation. For each ablation setting and benchmarking of VentWaveT against general-purpose TSFMs, performance metrics were estimated using 1,000 patient-level bootstrap resamples. Within each bootstrap resample, performance was calculated for both the pretrained and comparison models, and paired performance differences were computed. Distributions of paired performance differences are reported as means with 95% confidence intervals (CIs), together with medians and interquartile ranges (IQRs). Reconstruction performance was evaluated using patient-level block bootstrap resampling with Pearson correlation and root-mean squared error (RMSE) between the original and reconstructed waveforms. Deep learning models were implemented in Python 3.11 using the PyTorch framework (version 2.5.1)[20] and trained on NVIDIA graphics processing units (GPUs). Data preprocessing and downstream machine learning analyses were performed using Scikit-learn (version 1.8.0)[21]. Statistical analyses and data visualization were performed in R (version 4.4.1).

### Results

## The Ventilation Waveform Transformer model captures ventilator mode information

In this pilot feasibility study, we developed a transformer-based representation learning framework using ventilator pressure and flow waveforms (Fig. 1). Following self-supervised pretraining on randomly sampled 15-second waveform segments, respiratory-cycle embeddings were extracted from pressure and flow waveforms and evaluated using multinomial logistic regression models for downstream ventilator mode classification.

Across 1,000 mode-stratified patient-level block bootstrap resamples, the mean accuracy ranged from 0.863 to 0.975 across ventilation mode settings (Fig. 2). Pressure Control Ventilation (PCV) showed the lowest classification performance with a mean accuracy of 0.863 (95% CI, 0.773, 0.933). Mean F1-scores ranged from 0.587 to 0.851 across ventilation modes. Proportional Assist Ventilation (PAV) was the most challenging mode to classify, with a mean F1-score of 0.587 (95% CI: 0.390, 0.735). Of the 1000 bootstrap samples, 79 bootstrap samples for VC were excluded based on the prespecified true-label proportion threshold, whereas all 1000 samples were kept for the other ventilation modes.

We further projected the pretrained embeddings into a two-dimensional UMAP space to visualize the organization of the learned respiratory representations (Supplementary Figure E1 A-E). Illustrative waveforms from neighboring regions exhibited similar pressure and flow morphologies. Embedding-feature attribution maps highlighted the inspiratory pressure rise, inspiratory plateau, inspiratory-to-expiratory transition, and expiratory flow profile as the primary waveform regions contributing to the construction of mode-informative latent representations.

## The Ventilation Waveform Transformer model recovers ventilator and respiratory characteristics from representation learning

To evaluate the applicability for other downstream prediction tasks, we applied linear regression models on frozen embeddings to predict RR on a breath-by-breath basis. The mean MAE ranged from 1.620 to

2.432 breaths/min across ventilation modes (Fig. 3A). Volume Control Ventilation (VCV) showed the lowest performance, with a mean MAE of 2.432 breaths/min (95% CI: 0.602, 3.922). The mean Pearson correlation coefficient ( $\rho$ ) ranged from 0.798 to 0.958, and VCV suffered most, with a mean $\rho$ of 0.798 (95% CI: 0.651, 0.885) (Fig. 3B).

We further applied the pre-trained embeddings to predict PEEP for each breath. The mean MAE ranged from 0.283 to 0.634 $\mathrm{cmH_2O}$ across ventilation modes, while Volume Control Ventilation (VCV) showed the lowest performance, with a mean MAE of 0.634 $\mathrm{cmH_2O}$ (95% CI: 0.228, 0.967) (Fig. 3C). The mean Pearson correlation coefficient ( $\rho$ ) ranged from 0.776 to 0.943, and PAV suffered the most, with a mean $\rho$ of 0.776 (95% CI: 0.350, 0.985) (Fig. 3D).

## Ablation experiments

To assess whether pretrained embeddings capture physiologically relevant respiratory information, we compared VentWaveT embeddings with random embeddings generated from feature-wise Gaussian distributions parameterized by the means and standard deviations of the pretrained latent representations.

For ventilator mode classification, pretrained VentWaveT embeddings consistently outperformed random embeddings across most ventilator modes. Significant improvements in classification accuracy were observed for CPAP, PCV, PS, and VCV (Supplementary Figure E2). The largest improvement was observed for PS, with a mean accuracy increase of 0.405 (95% CI, 0.265, 0.528). Similarly, F1-scores were substantially improved using pretrained embeddings, with mean differences ranging from 0.230 to 0.851 across ventilator modes.

We further assessed the linear separability of the pretrained embeddings by comparing ventilator mode classification performance using multinomial logistic regression, a shallow MLP, and XGBoost models. For VCV, multinomial logistic regression achieved significantly higher accuracy than the shallow MLP, with a mean paired accuracy difference of 0.05 (95% CI: 0.018, 0.099) (Supplementary Figure E3-A). Similarly, F1 scores for VCV were higher with multinomial logistic regression, with a mean paired difference of 0.407 (95% CI: 0.222, 0.638). When comparing with XGBoost models for VCV, multinomial logistic regression achieved a higher magnitude of accuracy score (a mean paired difference: 0.105, 95% CI: 0.035, 0.198) and F1 score (a mean paired difference: 0.835, 95% CI: 0.651, 0.952) (Supplementary Figure E3-B). Similar results were observed for PAV.

For respiratory physiology prediction, pretrained embeddings also showed significantly superior performance for both RR and PEEP estimation compared with random embeddings. Across ventilator modes, pretrained embeddings reduced RR prediction error (MAE) by 2.806 to 4.940 breaths/min and increased Pearson correlation coefficients ( $\rho$ ) by 0.783 to 0.962 (Supplementary Figure E4-A and B). For PEEP estimation, pretrained embeddings reduced MAE by 1.419 to 3.166 $\mathrm{cmH_2O}$ and increased $\rho$ by 0.765 to 0.947 (Supplementary Figure E4-C and D).

## Comparable mode classification against TSFMs

To evaluate the quality of embeddings, VentWaveT was benchmarked against general-purpose TSFMs, MOMENT and Chronos-2, for ventilator mode classification using multinomial logistic regression. The mean of paired differences of accuracy across ventilation modes between VentWaveT and MOMENT was not statistically different (mean: -0.020, 95% CI: -0.056, 0.013). This pattern was consistent across other metrics (Supplementary Table E2, Supplementary Figure E5-A). Similarly, the paired performance differences between VentWaveT and Chronos-2 were not statistically different (Supplementary Table E2, Supplementary Figure E5-B).

## Waveform reconstruction based on unseen respiratory embeddings

To evaluate the reconstruction performance of waveforms based on unseen respiratory embeddings, patient-level bootstrap resampling was performed for Pearson correlation and RMSE between reconstructed waveforms from randomly shifted waveforms and the corresponding original waveforms. The median of Pearson correlation was 0.801 (95% CI: 0.727, 0.870) for pressure waveforms and 0.783 (95% CI: 0.709, 0.838) for flow waveforms between the reconstructed and original waveforms (Fig. 4A). The median RMSE was 2.757 $cmH_2O$ (95% CI: 2.106, 3.579) for pressure waveforms and 15.301 L/min (95% CI: 13.492, 18.187) for flow waveforms. Illustrative reconstructed waveforms from different ventilation modes are shown in Fig. 4B.

### Discussion

In this pilot study, we developed VentWaveT, a self-supervised representation learning framework based on a one-dimensional convolutional neural network and transformer architecture to extract physiological and mechanistic respiratory information from ventilator-derived pressure and flow waveforms. Across multiple downstream tasks, including ventilator mode classification and prediction of respiratory physiological variables, our results demonstrated the feasibility of extracting physiological and mechanistic information from pressure and flow ventilator waveforms without requiring manual annotation during pretraining.

Unlike conventional approaches that focus on handcrafted waveform features or task-specific prediction using a breath-cycle basis[7–11], we trained VentWaveT using randomly sampled 15-second waveform segments extracted from longitudinal ventilator recordings. This segmentation-independent strategy enables the model to learn respiratory representations directly from arbitrary waveform snapshots without requiring breath-level segmentation or manual identification of inspiratory and expiratory boundaries. As a result, respiratory embeddings can be generated from short intervals of routinely collected ventilator data, potentially supporting near real-time respiratory state characterization, bedside monitoring, and future clinical decision-support applications.

The model was pretrained using 255,355 breaths spanning 197.1 hours of mechanical ventilation across 115 patients, substantially smaller than typical modern foundation model training datasets. In our sample-size experiments, reconstruction performance improved markedly when the training cohort increased from 20 to 80 patients, after which performance plateaued. One possible explanation is that ventilator waveforms are generated by a relatively constrained physiological system. While patient characteristics vary considerably, the underlying respiratory mechanics and ventilator control strategies produce waveform patterns that are highly structured and repetitive. Consequently, respiratory representations may be learned from a relatively modest number of patients compared with foundation models developed for more heterogeneous data modalities such as natural language or genomic data[16].

We further evaluated the quality of the learned respiratory embeddings through exploratory ablation studies and benchmarking against general-purpose time-series foundation models. First, linear logistic regression performed favorably compared with the evaluated nonlinear classifiers, including XGBoost and MLPs, for ventilator mode classification. This finding suggests that VentWaveT organizes respiratory information into a latent space that is largely linearly separable, indicating that clinically relevant waveform characteristics are effectively encoded during self-supervised pretraining. Further studies using larger ventilation waveform datasets and systematic optimization of nonlinear models are warranted to characterize the structure of the learned latent space. Second, mode classification performance with VentWaveT was not largely, statistically different from that of two state-of-the-art general-purpose time-series foundation models[18,19], despite being pretrained on substantially fewer patients and only ventilator waveform data. Token averaging was applied consistently across models to obtain the embeddings, while PCA was applied to the general-purpose time-series foundation models to match the dimension of the VentWaveT representation for downstream evaluation. Although PCA provides a linear dimensional reduction, mode-specific information necessary to predict ventilation mode may have been lost during dimensionality reduction. Nevertheless, this approach enabled comparison of equally sized representation using the same downstream models. Together, these findings suggest that respiratory waveforms contain structured physiological information to support domain-specific representation learning.

Different ventilator modes impose distinct physiological and mechanical constraints on waveform generation. To improve the interpretability of the learned representations, we generated embedding-feature attribution maps for representative waveforms. The attribution maps consistently highlighted waveform regions routinely examined by clinicians, including the inspiratory pressure rise, inspiratory plateau, inspiratory-to-expiratory transition, and expiratory flow morphology. Together with the ability of the pretrained embeddings to accurately identify ventilator modes and predict respiratory physiological variables, these findings suggest that VentWaveT captures clinically relevant waveform characteristics associated with respiratory characteristics and ventilator operation.

Patient–ventilator asynchrony remains one of the major challenges in mechanical ventilation[22,23]. VentWaveT provides a potential computational foundation for investigating asynchrony through representations learned from unannotated pressure and flow waveforms. Future studies could evaluate these representation embeddings for detecting patient–ventilator asynchronies, including ineffective triggering, double triggering, reverse triggering, and flow starvation. Longitudinal embedding trajectories may also enable quantification of the cumulative burden of asynchrony over time, providing an opportunity to investigate whether persistent patient–ventilator mismatch contributes to subsequent ventilator outcomes. Supervised annotations of asynchrony events will be required for downstream model development and validation. Integration with clinical and molecular data modalities may facilitate development of next-generation, personalized ventilator monitoring system, capable of continuous assessment of patient–ventilator interaction, early identification of patients at risk for prolonged ventilator dependence, and dynamic adaptation of ventilation strategies to patient-specific physiological needs.

Several limitations should be acknowledged. First, this was a pilot study conducted using data from a limited number of patients and ventilator settings. Second, only a subset of respiratory variables was evaluated as downstream tasks. Third, while the learned embeddings demonstrated strong predictive performance, the physiological interpretation of individual latent dimensions remains unclear. Additional studies incorporating robust benchmarking and ablation control designs are needed to evaluate representation stability across institutions, ventilator manufacturers, patient populations, and patterns of patient-ventilator asynchrony events.

## Conclusion

In this pilot feasibility study, we developed VentWaveT, a self-supervised representation learning framework that learns respiratory representations directly from ventilator pressure and flow waveforms without requiring manual annotations. The learned embeddings supported multiple downstream tasks, including ventilator mode classification and prediction of respiratory physiological variables, and preserved sufficient physiological information to reconstruct ventilator waveforms from previously unseen patients. Beyond the tasks evaluated in this study, VentWaveT introduces ventilator waveforms as a useful data modality in critical care informatics and establishes a computational framework for future investigations of patient–ventilator asynchrony, respiratory state monitoring, optimization of liberation from mechanical ventilation, and multimodal integration with clinical, physiological, and molecular data. Collectively, our results support the feasibility of a ventilator waveform representation learning and highlight the potential role of ventilator waveform foundation models in next-generation respiratory monitoring and precision mechanical ventilation management in critical care.

## Declarations

Author contributions

S.K. and R.K. conceptualized the research direction, developed methodology, performed formal analyses, and wrote the original manuscript. V.K., T.O., M.V.P., K.L.H., S.M.A., and R.K. supervised, reviewed, and revised the manuscript. All authors approved the final version of the manuscript.

Declaration of interests

The authors declare no competing interests.

**Funding**

S.K. was supported by the National Institutes of Health (NIH) under Award Number R21GM148931 and R01HL170175. RK was supported by the NIH under Award Numbers R01GM139967, R21GM151703, R21GM148931, and R01HL170175.

Data sharing statement

This study did not generate new materials. Data used in this analysis was publicly available. R and python scripts used for data preprocessing and analysis are available from https://github.com/Kamaleswaran-Lab/ventwave_transformer.

Ethical approval statement

The datasets used in this study are publicly available and de-identified. All study procedures were conducted in accordance with the ethical principles of the Declaration of Helsinki.

## Figures

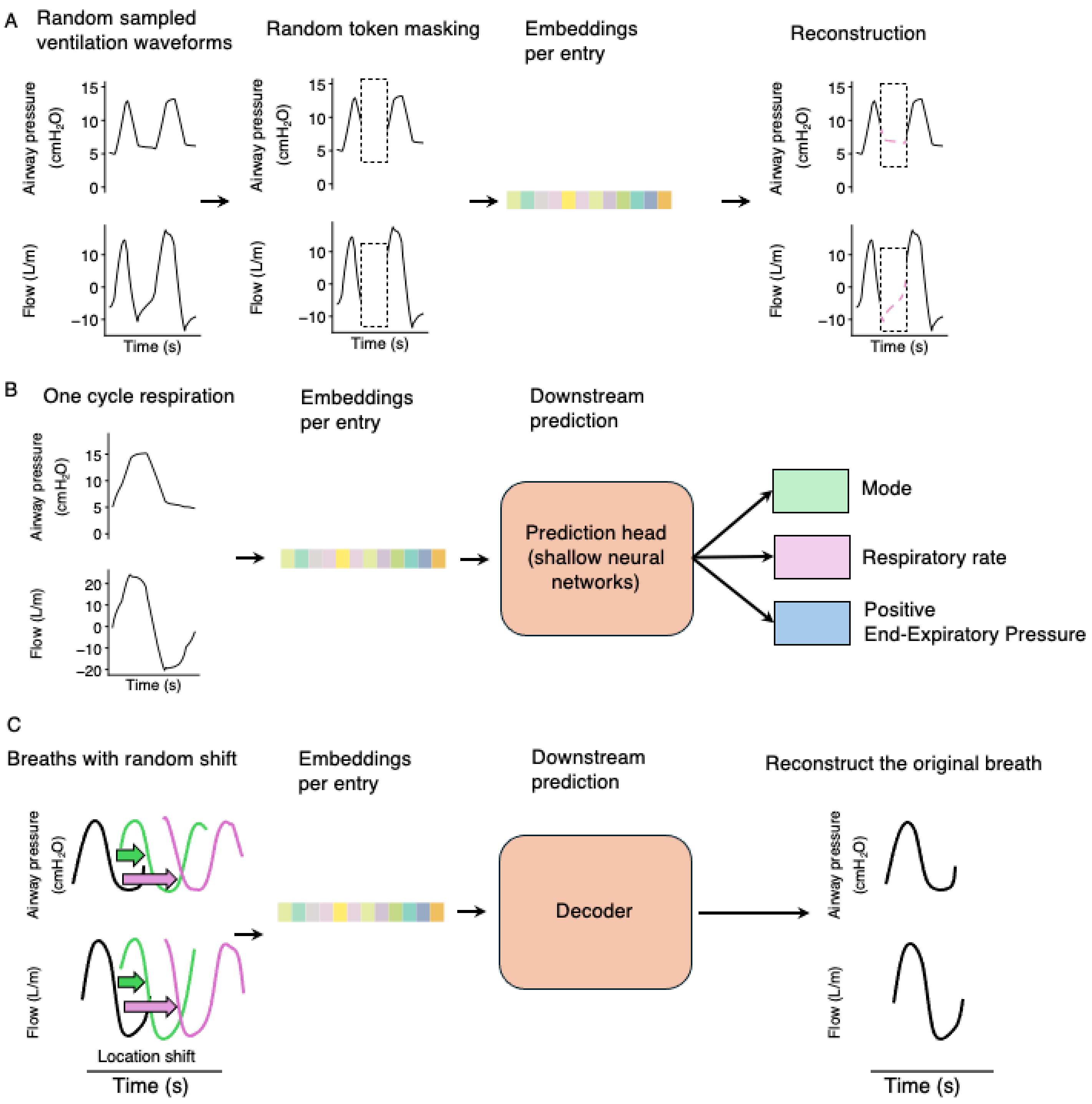

**Figure 1**

Overview of the Ventilator Waveform Transformer (VentWaveT). (A) Self-supervised pretraining of VentWaveT using airway pressure and flow waveforms sampled from continuous mechanical ventilation recordings. The model learns latent respiratory representations without requiring manual annotations. (B) Downstream evaluation using frozen embeddings and linear prediction heads for ventilator mode classification and prediction of respiratory physiological variables. (C) Transformer-based decoding of respiratory embeddings into ventilator waveforms. Reconstruction was evaluated by decoding embeddings extracted from randomly shifted waveforms and comparing the reconstructed waveforms with the corresponding original, non-shifted waveforms.

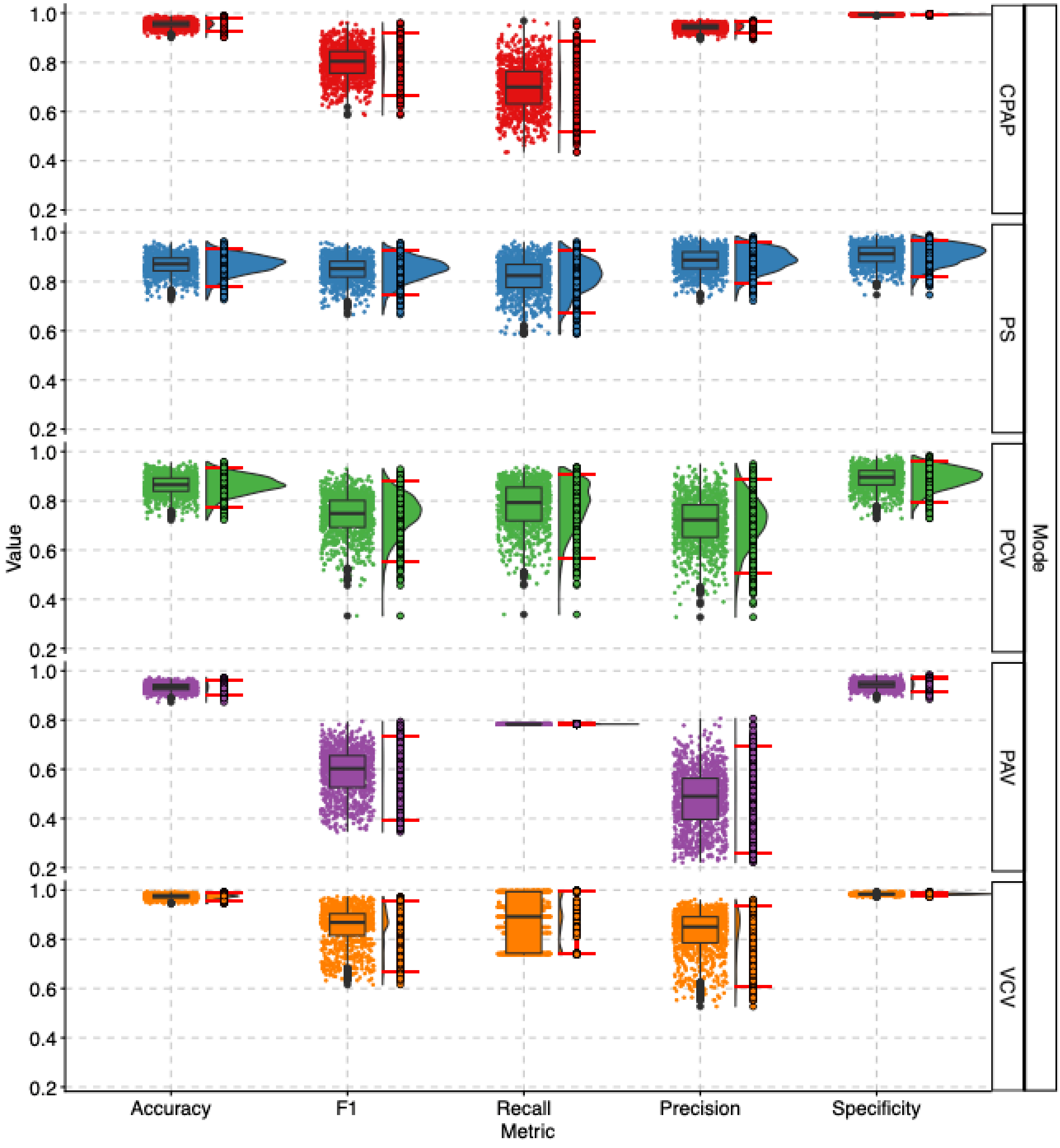

### Figure 2

VentWaveT accurately recovers ventilator mode settings from waveform-derived embeddings. Frozen embeddings generated by VentWaveT were used for ventilator mode classification using multinomial logistic regression models. Performance was evaluated using accuracy, F1-score, recall, precision, and specificity. Distributions were estimated using 1,000 patient-level bootstrap resamples and are presented as means with 95% confidence intervals, medians, and interquartile ranges.

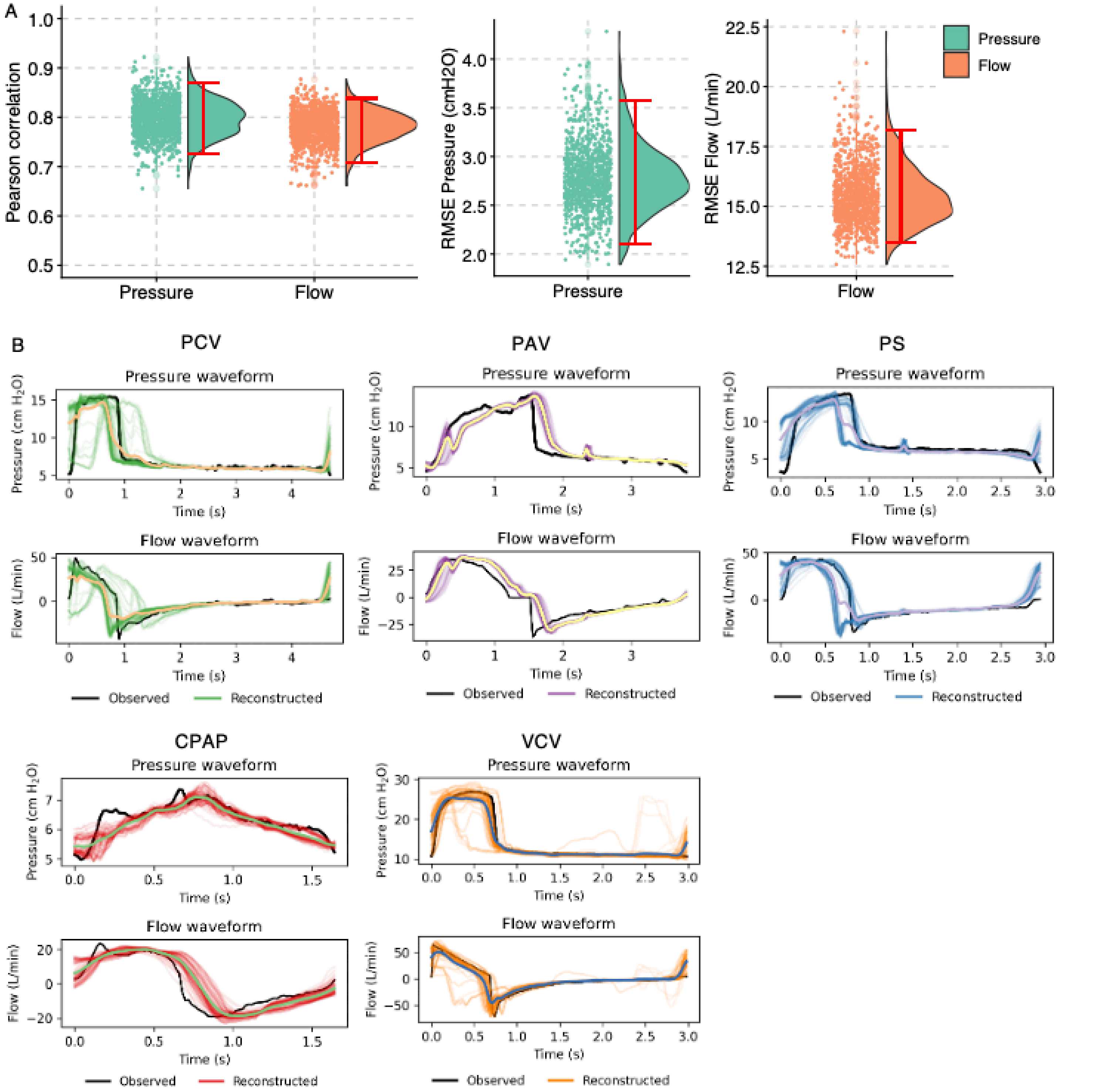

### Figure 3

VentWaveT embeddings recover ventilator and respiratory characteristics. Frozen embeddings generated by VentWaveT were used to predict respiratory rate (RR) and positive end-expiratory pressure (PEEP) using linear regression models. (A) Mean absolute error (MAE) for RR prediction. (B) Pearson correlation coefficient  for RR prediction. (C) MAE for PEEP prediction. (D) Pearson correlation coefficient  for PEEP prediction. Performance distributions were estimated using 1,000 patient-level bootstrap resamples and are presented as means with 95% confidence intervals, medians, and interquartile ranges.

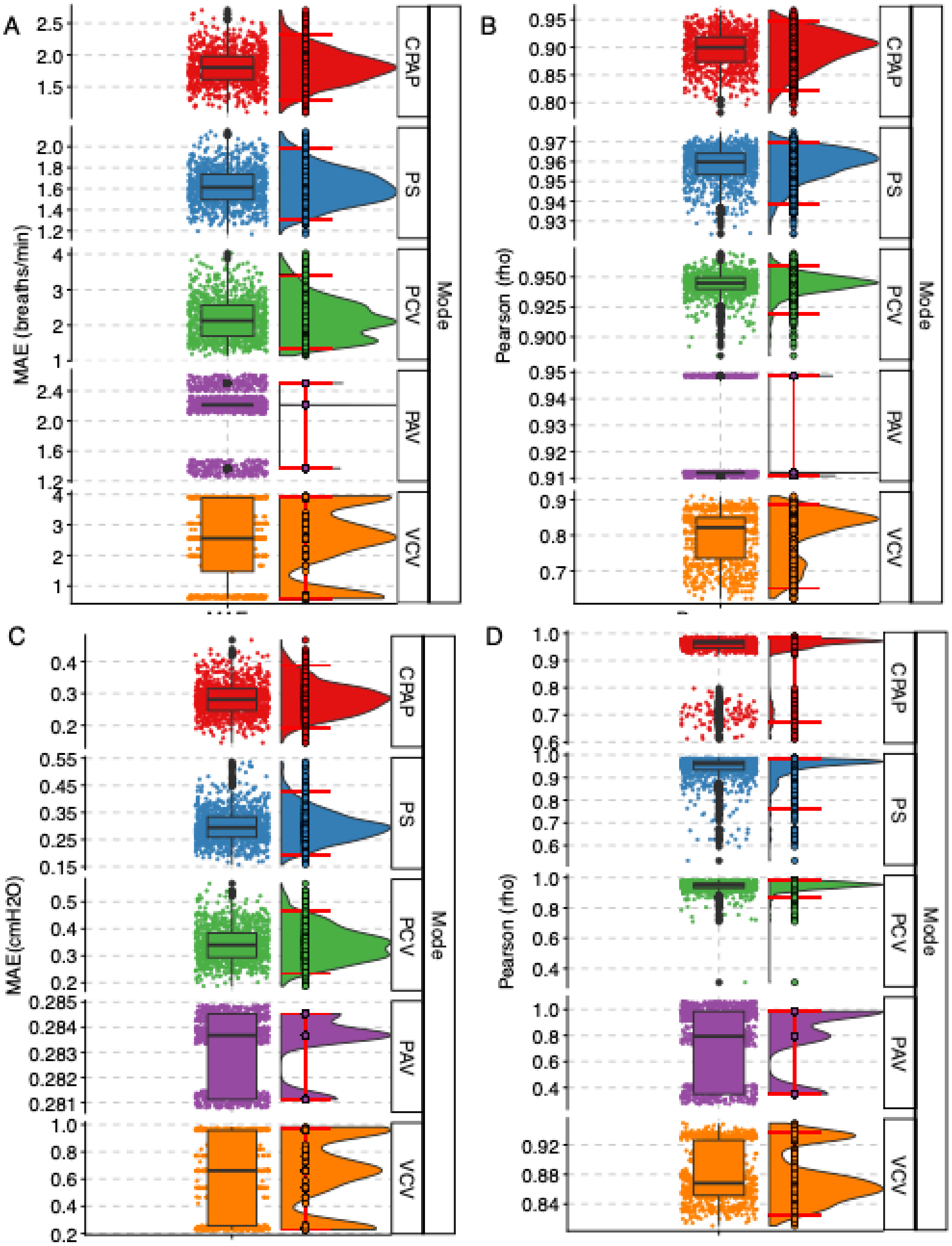

**Figure 4**

VentWaveT reliably reconstructs ventilator waveforms from unseen respiratory embeddings. Respiratory embeddings were extracted from randomly shifted waveforms and decoded into ventilator waveforms using the VentWaveT decoder. Reconstruction performance was evaluated by comparing the reconstructed waveforms with the corresponding original, non-shifted waveforms using Pearson correlation (A, left) and root mean squared error (RMSE; A, right). Performance metrics and 95% confidence intervals were estimated using 1,000 patient-level block bootstrap resampling. (B) Selected reconstructed waveforms across ventilation modes. For each panel, a black line denotes the original, non-shifted waveforms. Thin lines represent reconstructions from randomly shifted waveforms, and the bold line represents their mean.

### Supplementary Files

This is a list of supplementary files associated with this preprint. Click to download.

- ventilationrepresentationlearningsup020261009.docx
